# Relationship between patellofemoral finite helical axis and femoral trans-epicondylar axis using a static magnetic resonance-based methodology

**DOI:** 10.1186/s13018-021-02328-2

**Published:** 2021-03-24

**Authors:** Zhenguo Yu, Hong Cai, Bin Yang, Jie Yao, Ke Zhang, Hua Tian, Zhongjun Liu

**Affiliations:** 1 Department of Orthopedics, Peking University Third Hospital, No.49 North Garden Road, Haidian District, Beijing, 100191 China; 2 Department of Orthopedics, Peking University International Hospital, Life Park 1, Zhongguancun Life Science Park, Changping District, Beijing, 102206 China; 3 Key Laboratory for Biomechanics and Mechanobiology of Ministry of Education, School of Biological Science and Medical Engineering, Beijing Advanced Innovation Centre for Biomedical Engineering, Beihang University, No.37 Xueyuan Road, Haidian District, Beijing, 100191 China

**Keywords:** Patellofemoral joint, Finite helical axis, Trans-epicondylar axis, Knee

## Abstract

**Background:**

To manage patellofemoral joint disorders, a complete understanding of the in vivo patellofemoral kinematics is critical. However, as one of the parameters of joint kinematics, the location and orientation of the patellofemoral finite helical axis (FHA) remains unclear. The purpose of this study is to quantify the location and orientation of the patellar FHA, both in vivo and non-invasively at various flexion angles, and evaluate the relationship of the FHA and the trans-epicondylar axis (TEA).

**Methods:**

The magnetic resonance (MR) images of 18 unilateral knees were collected at full extension, 30°, 60°, 90°, and maximum angle of knee flexion. Three-dimensional models of the knee joint at different flexion angles were created using the MR images, and then used to calculate the patellar tracking and FHA with a spline interpolation algorithm. By using a coordinate system based on the TEA, the FHA tracking was quantified. Six parameters concerning the location and orientation of the patellar FHA were analysed.

**Results:**

The average patellar FHA drew an L-shaped tracking on the midsagittal plane moving from the posteroinferior to the anterosuperior side of the TEA with knee flexion. Before 90° flexion, the patellar rotational radius decreased slightly, with an average value of 5.65 ± 1.09 cm. During 20° to 90° knee flexion, the average angle between the patellar FHA and the TEA was approximately 10° and that between the FHA and the coronal plane was maintained at about 0°, while that between the FHA and the level plane fluctuated between − 10° and 10°.

**Conclusions:**

This study quantitatively reported the continuous location and direction of the patellar FHA during knee flexion. The patellar FHA was close to but not coincident with the femoral TEA both in location and orientation, and the patellar rotational radius decreased slightly with knee flexion. These findings could provide a clear direction for further studies on the difference in patellofemoral FHA among various types of patellofemoral disorders, and provide a foundation for the application of FHA in surgical evaluation, preoperative planning and prosthesis design, thereby assisting in the diagnosis and treatment of patellofemoral disorders.

**Supplementary Information:**

The online version contains supplementary material available at 10.1186/s13018-021-02328-2.

## Introduction

Patellofemoral disorders have a high incidence rate and are challenging to manage [1, 2]. To explore the pathogenesis and improve the therapeutic efficacy of these disorders, it is crucial to fully understand the in vivo patellofemoral kinematics. Patellar tracking and the finite helical axis (FHA) are both parameters of patellofemoral kinematics. Patellar maltracking is a common concern in the management of patellofemoral disorders [3, 4]. However, high dependence on the coordinate system of patellar tracking leads to ambiguity in the consensus of the definition of normal tracking [5, 6], and therefore is not accurate enough when evaluating patellofemoral kinematics with patellar tracking.

Patellar FHA refers to the central axis of patellar rotation during knee flexion. Unlike patellar tracking, the calculation of FHA is independent of the coordinate system, which is embedded in the ‘moving body’. In addition, the FHA can be directly applied to analyse the moment of the muscles to which the biomechanics of joint is related [7]. In the tibiofemoral joint, a close relationship between the FHA and the femoral trans-epicondylar axis (TEA) is demonstrated [7, 8]. In consideration for the coupled motion of the tibiofemoral and patellofemoral articulations, a similar relationship between the patellar FHA and the TEA might exist. Iranpour et al. [9] stated that the patella moved in a circle around the trochlear axis almost parallel to the TEA. Coughlin et al. [10] showed that the patellar motion followed a nearly perfect circular arc in the midsagittal plane of the femur, with the origin of this arc at 9.6 mm anterior and 11.6 mm proximal to the femoral TEA.

However, there are no descriptions about the location and orientation of the continuous patellar FHA at various knee angles, which is typically displayed as a series of straight lines encompassed by arcuate patellar motion. Thus, the primary purpose of this study was to quantify the location and orientation of the patellar FHA and relate it to the femoral TEA with a non-invasive and in vivo methodology based on static magnetic resonance (MR), which can provide a new starting point in discovering the pathogenesis and improving the therapeutic effect of patellofemoral disorders.

## Methods

### Study participants

Eighteen healthy subjects participated in this study (Table 1). The study was approved by the Ethics Committee of Peking University International Hospital. All subjects received an oral and written explanation of the study and signed the informed consent form. None of the subjects had a history of anterior knee pain, clinically diagnosed knee pathology, previous knee joint surgery, or contraindications to MR scanning.

**Table 1:** Demographic characteristics of the subjects

Characteristics	Value
Gender (male/female)	9/9
Age^a^ (years)	26.6 ± 4.9
Height^a^ (cm)	170.0 ± 6.7
Weight^a^ (kg)	61.1 ± 9.2
BMI^a,b^ (kg/m^2^)	21.1 ± 2.5

^a^The values are given as the mean and the standard deviation

^b^*BMI* body mass index

### MR scanning

**Fig. 1:**
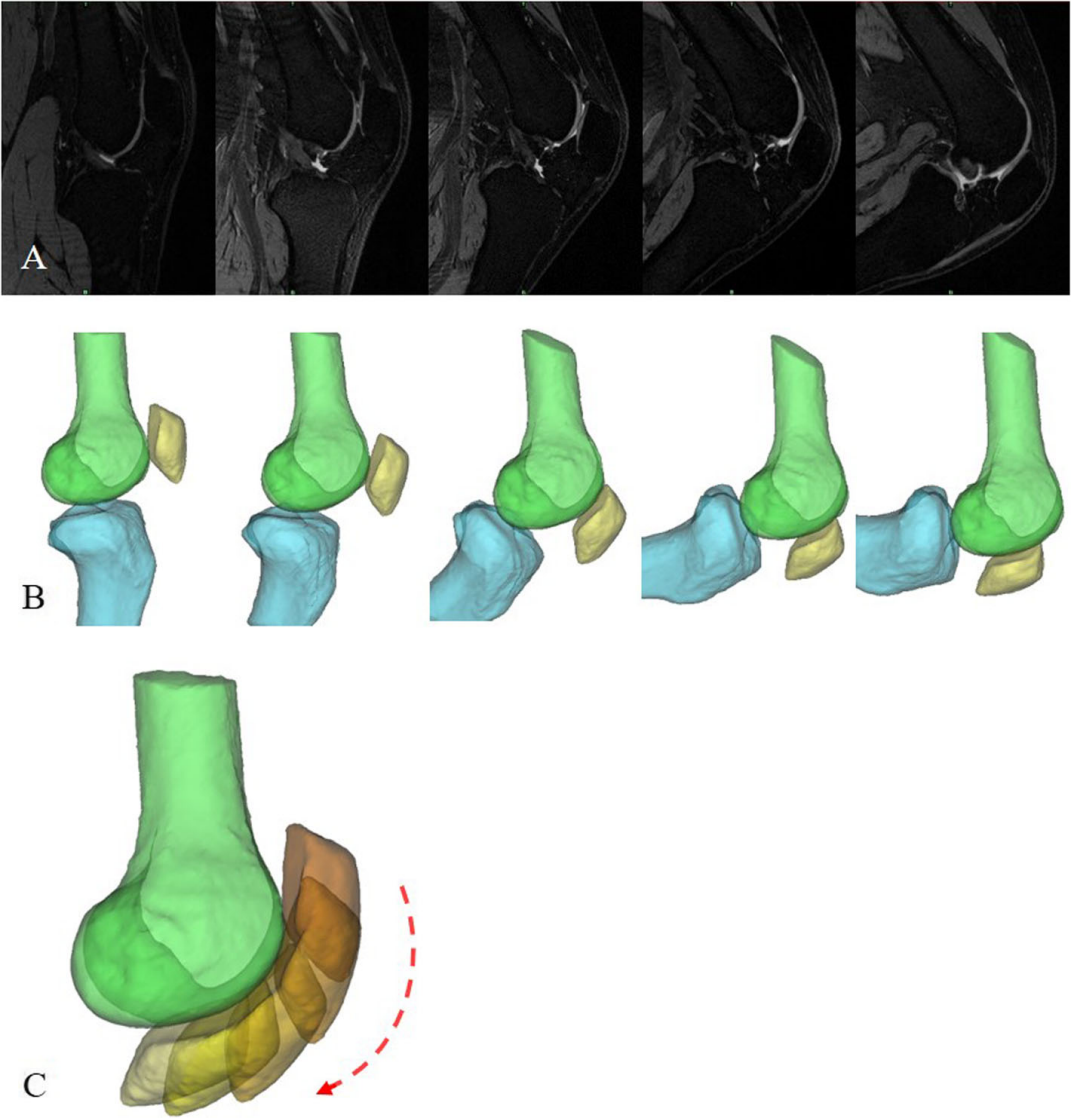
Geometric reconstruction of the knee joint. **a** The knee joint was scanned with the MR machine at full extension and at 30°, 60°, 90°, and maximum angle of knee flexion. **b** The 3D models of the femur, patella, and tibia were developed with the medical image processing software, Mimics. **c** Patellar tracking was calculated from the above knee models at five angles of knee flexion

The unilateral knee of each subject was scanned with the MR machine (Siemens/Verio 3.0T, Germany) at full extension and at 30°, 60°, 90°, and maximum angle of knee flexion. The subjects were positioned laterally. A thermoplastic knee fixator was used to keep the target knee at the required angle and immobilised during scanning. The following scanning parameters were used: fat-suppression T2-weighted image; slice increment = 0.999 mm; slice thickness = 1 mm; resolution = 512pxl × 512pxl; pixel size = 0.352 mm. The MR images of the knee’s sagittal section at five angles of knee flexion are shown in Fig. 1a.

### Geometric reconstruction and register

Based on the MR images, three-dimensional (3D) models of the femur, patella, and tibia were developed with the medical image processing software Mimics (version 16.0 Materialise, Inc., Belgium) (Fig. 1b). The patella and tibia of each position were registered to the femur models by applying the inverse engineering software, Rapidform (version 2006, 3D Systems, Inc., Korea), and placing the femur models at various flexion angles in a fixed position (Fig. 1c). The knee flexion angles were recalculated with reconstructed models. The process of knee flexion was divided into three stages: early stage (0°–45°), middle stage (45°–90°), and late stage (> 90°).

### Calculation of patellar tracking and FHA

Continuous patellar tracking was calculated with the knee models at five knee flexion angles using order-three spline algorithm. This calculation method was developed in our previous studies [11, 12], and its accuracy to reconstruct continuous patellar motion was verified with a motion capture system [11]. Then, using the Chasles’ theorem, the FHA of the patellar motion was derived from the patellar tracking with 1° increments in the knee flexion angle; the motion process of the patellar FHA during knee flexion is shown in Additional file 1. In addition, the calculation formulas are shown in Additional file 2.

### Configuration of coordinate system

**Fig. 2:**
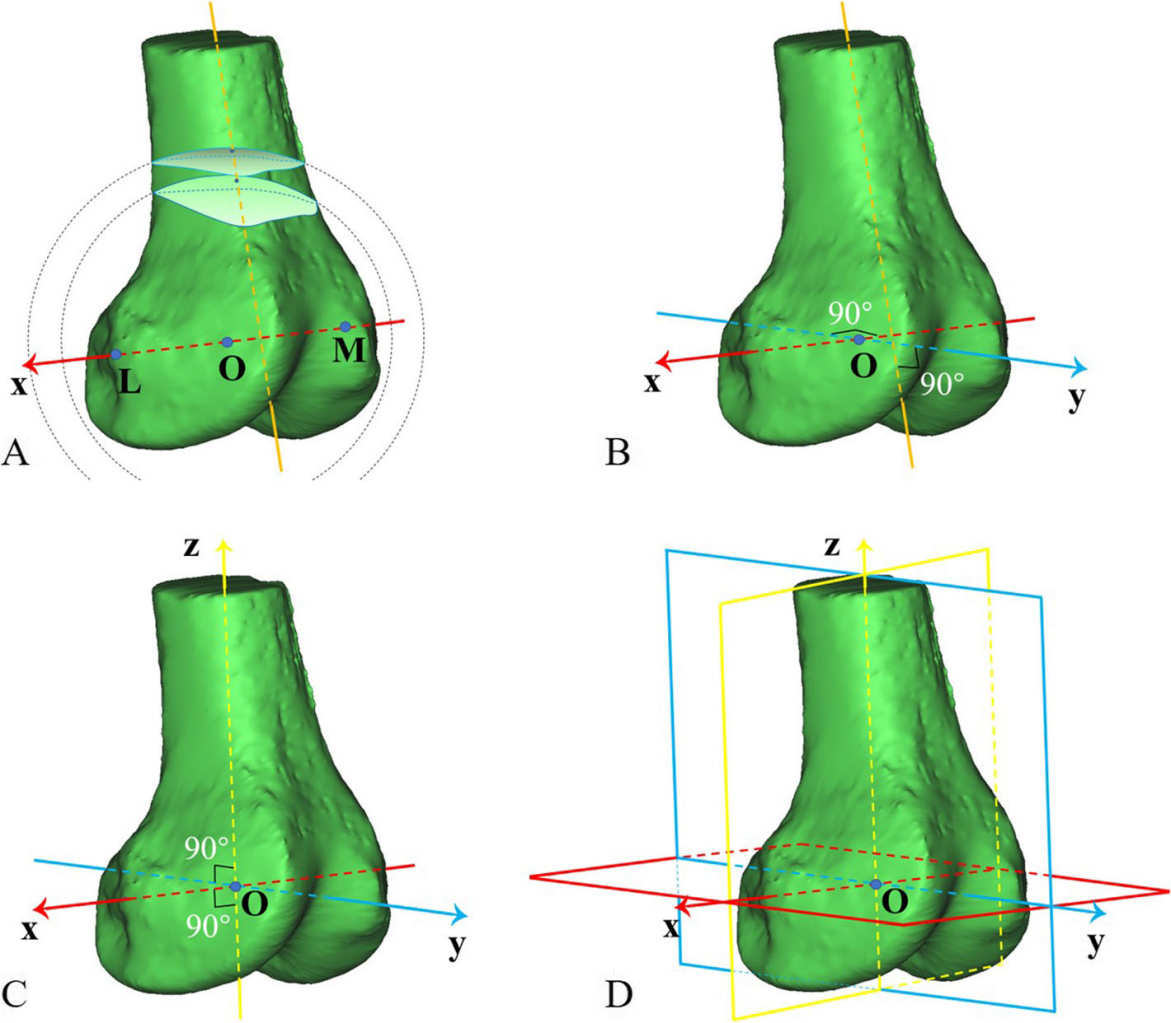
A coordinate system was established based on the femur. **a** The fovea of the medial epicondyle (point M) and the highest point of the lateral epicondyle (point L) were selected to form the TEA, which was defined as the x-axis, with the midpoint of the TEA as the origin (point O); two virtual balls—with the origin as their centres and with 0.7 and 0.8 times of the TEA length as their radius respectively—were crossed with the femur to obtain two section surfaces, whose centroids were linked to determine the femoral shaft axis. **b** The *y*-axis was defined as the line passing through the origin and perpendicular to the TEA and the femoral shaft axis meanwhile. **c** The *z*-axis was perpendicular to the x-axis and y-axis through the origin. **d** Three planes were determined by the *x*-, *y*- and *z*-axes (red: level plane; yellow: coronal plane; blue: midsagittal plane)

A coordinate system based on the femur was then established (Fig. 2). First, the sulcus of the medial epicondyle (point M in Fig. 2) and the prominence of the lateral epicondyle (point L in Fig. 2) were selected to form the femoral TEA [13], defined as the *x*-axis, with the midpoint of the TEA as the origin (point O in Fig. 2) and the direction from medial to lateral as positive. Second, the *y*-axis was established as the line passing through the origin and perpendicular to the TEA and the femoral shaft axis, with the direction from posterior to anterior as positive. As shown in Fig. 2a, the femoral shaft axis was formed by two section centres of the femoral shaft, which were 0.7 and 0.8 times the TEA length away from the origin, respectively. Finally, the *z*-axis was perpendicular to the *x*-axis and *y*-axis through the origin, and the direction from distal to proximal was positive.

To evaluate the influence of the TEA determination on the subsequent calculation of patellar FHA parameters, the intra- and inter-rater intraclass correlation coefficients (ICCs) of these parameters were computed with the assistance of two clinical orthopaedic doctors who consistently determined the TEAs on the 18 knee MR images with an interval time of 2 weeks or more.

### Parameters of patellar FHA

**Fig. 3:**
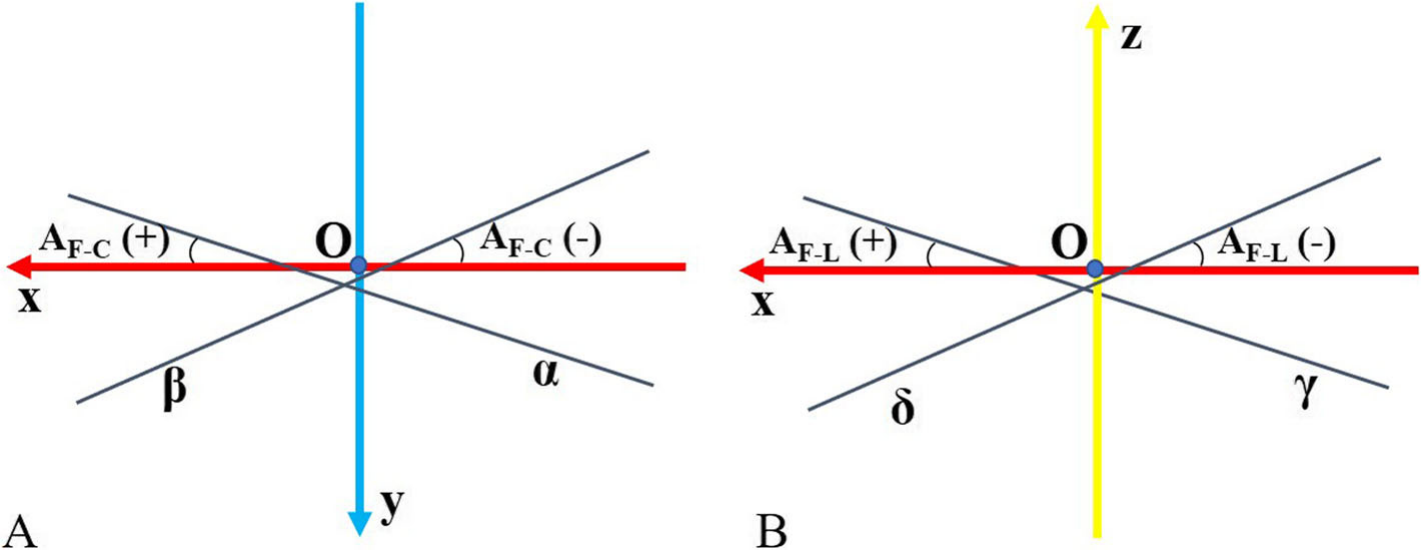
Definition of the orientation of the patellar FHA. **a** With respect to the angles between the patellar FHA and the coronal plane (A_F-C_), it was set positive when the FHA was located from posteromedial to anterolateral (line α) and negative when the FHA was located from posterolateral to anteromedial (line β). **b** With respect to the angles between the FHA and the level plane (A_F-L_), it was set positive when the FHA was located from superolateral to inferomedial (line γ) and negative when the FHA was located from superomedial to inferolateral (line δ)

To analyse the characteristics of FHA tracking, five parameters of location and orientation were quantified: (1) the intersection position (IP) between the FHA and midsagittal plane (IP_*y*_ = *y* coordinate of IP, IP_*z*_ = *z* coordinate of IP); (2) patellar rotational radius (PRR; the distance between the patellar centroid and its FHA) [9]; (3) spatial angles between the FHA and femoral TEA (A_F-T_); (4) angles between the FHA and coronal plane (A_F-C_; set positive when the FHA was located from posteromedial to anterolateral) (Fig. 3a); and (5) angles between the FHA and level plane (A_F-L_; set positive when the FHA was located from superolateral to inferomedial) (Fig. 3b). All parameters of the FHA location were normalised with the TEA length.

## Results

**Fig. 4:**
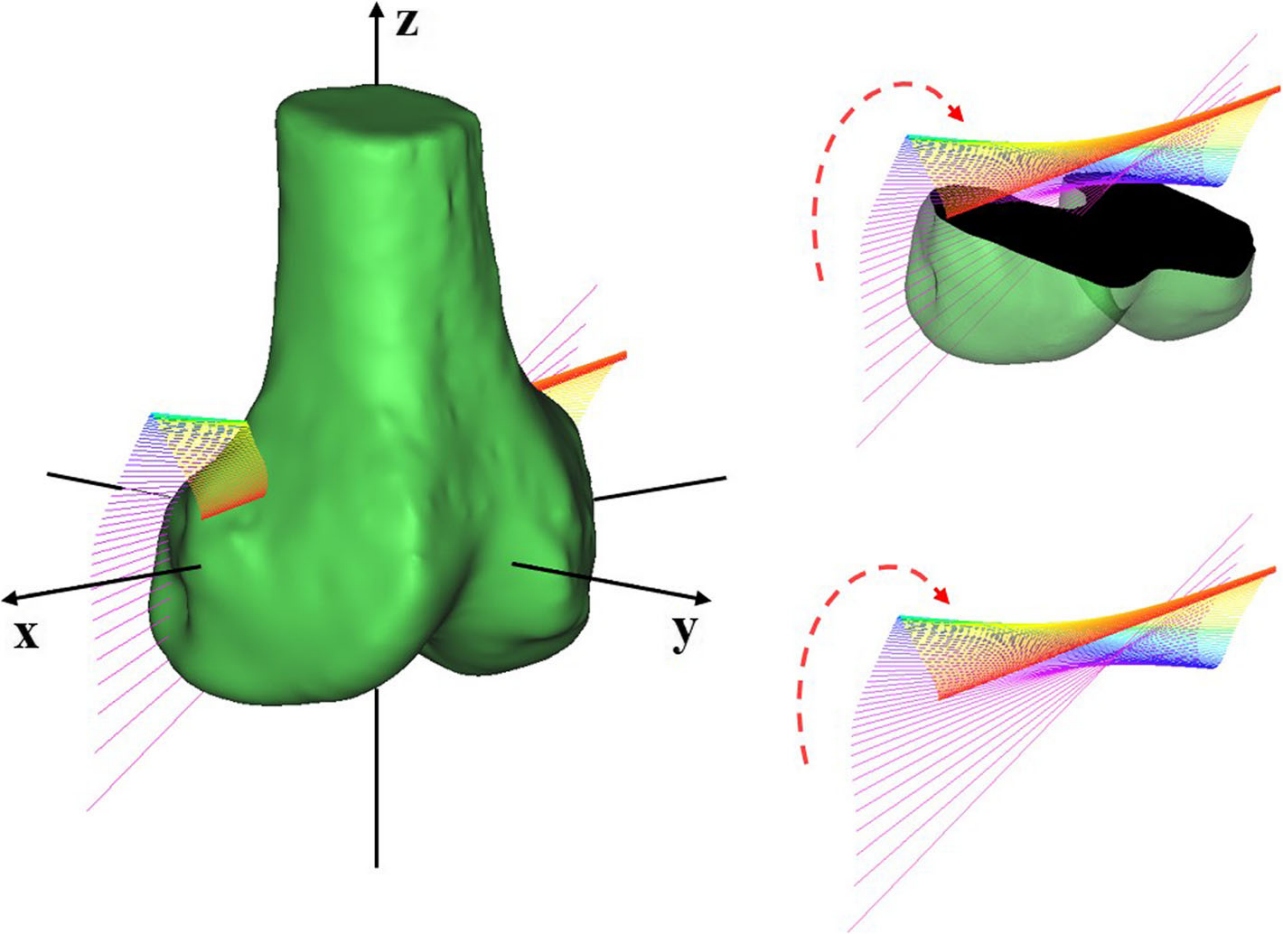
Three-dimensional tracking of the average patellar FHA. The average patellar FHA continuously changed with knee flexion, with a gradient of colour from purple to red

The parameter curves of the patellar FHA with knee flexion are shown in Additional file 3, and the 3D tracking of the average patellar FHA is depicted in Fig. 4. Intra- and inter-rater ICCs of all aforementioned parameters of the FHA exceeded 0.93 (Table 2). The average length of femoral TEA was 7.85 ± 0.53 cm (mean ± standard deviation).

**Table 2:** Intra- and inter-rater reliability of patellar FHA parameters

	Intra-Rater		Inter-Rater	
	ICC	(95% CI)	ICC	(95% CI)
IP_y_	0.997	(0.997 to 0.997)	0.995	(0.994 to 0.995)
IP_z_	0.989	(0.988 to 0.990)	0.941	(0.936 to 0.946)
PRR	0.997	(0.997 to 0.997)	0.996	(0.996 to 0.997)
A_F-T_	0.981	(0.980 to 0.983)	0.964	(0.960 to 0.967)
A_F-C_	0.979	(0.978 to 0.981)	0.965	(0.962 to 0.968)
A_F-L_	0.990	(0.989 to 0.991)	0.983	(0.981 to 0.984)

*A*_*F*-C_ angles between the FHA and coronal plane, *A*_*F*-*L*_ angles between the FHA and level plane, *A*_*F*-*T*_ spatial angles between the FHA and femoral TEA, *CI* confidence interval, *FHA* finite helical axis, *ICC* intraclass correlation coefficients, *IP* intersection position between FHA and midsagittal plane, *IP*_*y*_ *y* coordinate of IP, *IP*_*z*_ *z* coordinate of IP, *PRR* patellar rotational radius

### Tracking of patellar FHA

**Fig. 5:**
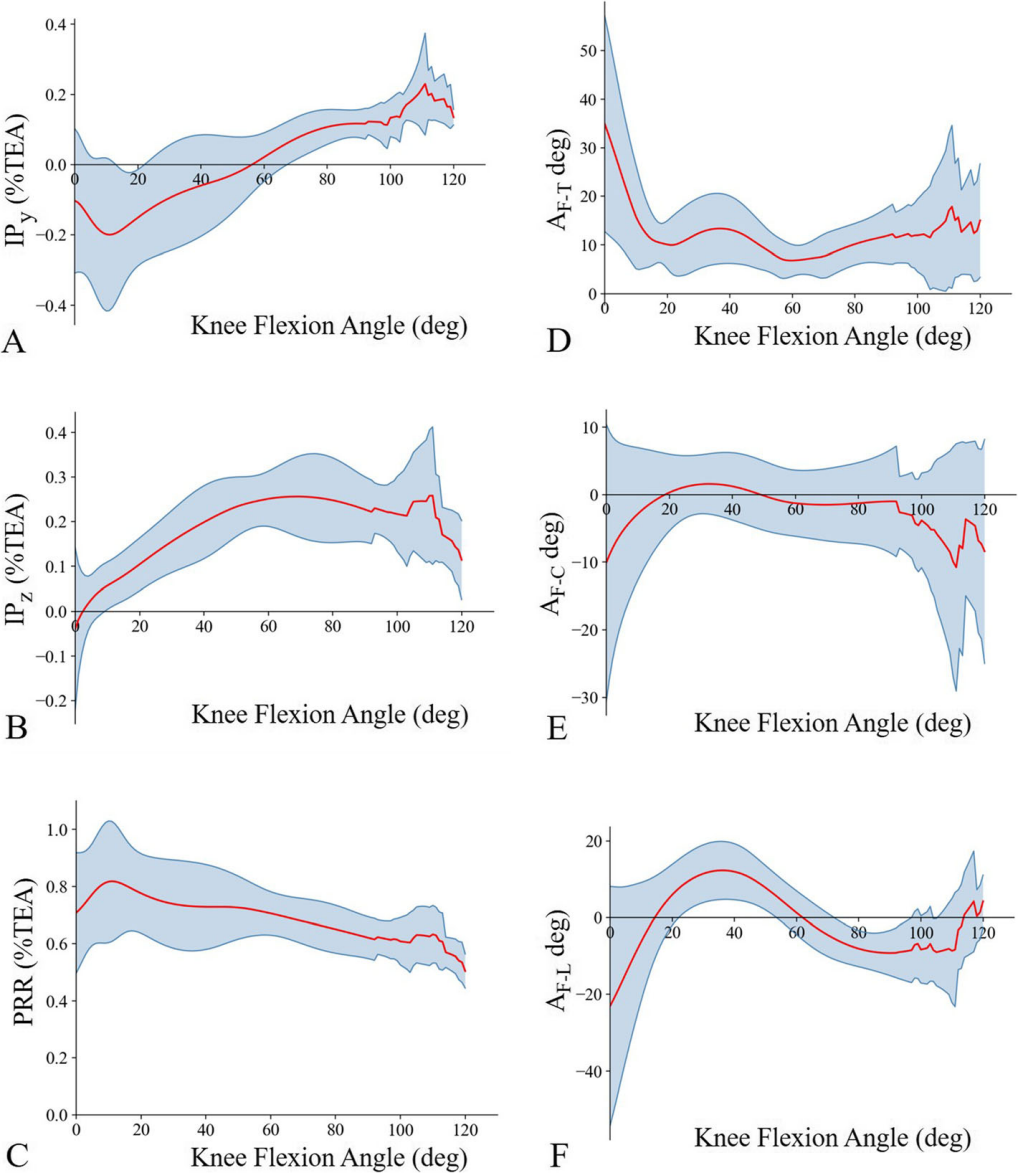
Average FHA parameters during knee flexion. **a** Average patellar FHA in the midsagittal plane moved from the posterior to the anterior side of the TEA. **b** Average patellar FHA in the midsagittal plane moved from the inferior to the superior side of the TEA. **c** Average patellar rotational radius increased during 0°–10° flexion and then decreased slightly. **d** Average angle between patellar FHA and the TEA fluctuated by about 10° during 20°–90° knee flexion. **e** Average angle between patellar FHA and coronal plane was maintained at about 0° during 20°–90° knee flexion. **f** Average angle between patellar FHA and level plane fluctuated between − 10° and 10°

**Fig. 6:**
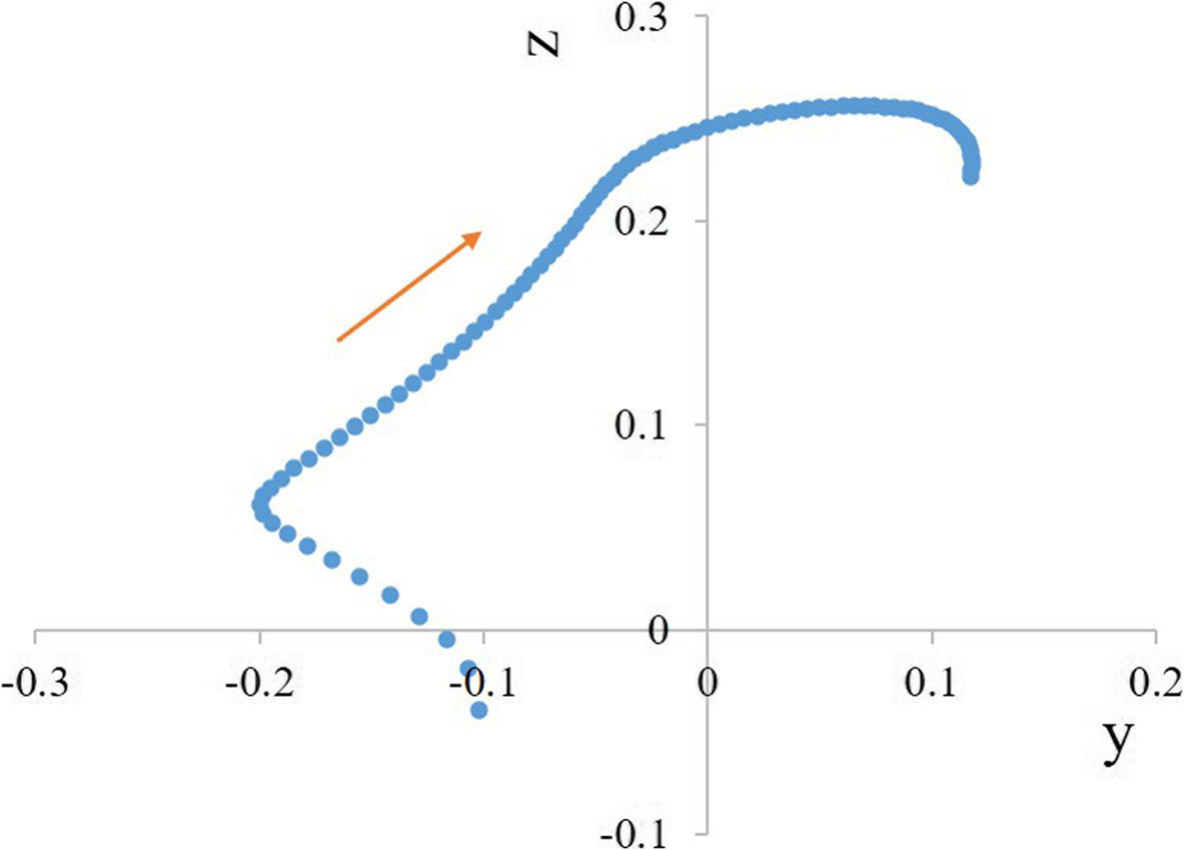
The intersection position of the FHA and the midsagittal plane. During 0°–90° knee flexion, the trajectory of the average intersection position was roughly L-shaped

With the knee flexing, the patellar FHA moved forward after shifting upward from the posterosuperior position of the TEA. Specifically, the average IP moved backward and upward from the position of 0.1 behind and 0.5 below the TEA, reaching positions of 0.2 behind and 0.05 above the TEA at 10° knee flexion. Subsequently, it moved forward and upward of the TEA, reaching 0.25 right above the TEA at 60° flexion. During 60°–90° knee flexion, the average IP continued to move forward to the position of 0.1 in front of the TEA (Figs. 5a, b and 6). During 0°–90° knee flexion, the trajectory of the average IP was roughly L-shaped (Fig. 6).

### PRR

In the first 20° of the early stage, the PRR fluctuated between 0.4 and 1.5, and varied in the range of 0.4–0.9 during 20°–45° flexion. In the middle stage, the PRR fluctuated within the range of 0.45–0.9. The average PRR increased during 0°–10° flexion and then gradually decreased (Fig. 5c). Regardless of the instantaneous change of PRR, the average PRR in the early and middle stages was 0.72 ± 0.14 times the TEA length (5.65 ± 1.09 cm).

### Orientation of patellar FHA

In the first 20° of the early stage, the A_F-T_ changed from 10°–80° to 0°–30°. During 20°–90° knee flexion, the A_F-T_ of 16/18 subjects fluctuated between 0° and 20°, and the average A_F-T_ was maintained at approximately 10° (Fig. 5d). In the late stage, A_F-T_ tended to increase as the knee flexed along with the increase of individual discrepancy.

In the first 20° of the early stage, the individual differences of A_F-C_ reached 80°, and the A_F-C_ of 16/18 subjects changed from (− 30°)–20° to (− 10°)–10°. During 20°–90° knee flexion, the A_F-C_ of every subject continued to fluctuate within the range of − 10°–10°. In the late stage, the patellar FHA of four subjects deviated from the coronal plane, and the others roughly maintained the previous direction. The average A_F-C_ was less than 5° in the early and middle stages, and less than 2° during 20°–90° of knee flexion (Fig. 5e).

In the early stage, the A_F-L_ of 17/18 subjects changed from (− 80°)–20° to 0°–20°, and the average A_F-L_ changed from − 25° to 10° (Fig. 5f). In the middle stage, this angle gradually changed to the range of (− 20°)–0°, with an average A_F-L_ changing to about − 10°, and the A_F-L_ in 13/18 subjects equalled 0° at 50°–70° knee flexion. In the late stage, the individual differences of A_F-L_ tended to be greater than that in the former stage.

## Discussion

Patellofemoral disorders are common and refractory [14]. Considering that joint kinematics is the mechanistic link between musculoskeletal anatomy and joint function, a complete understanding of the physical patellofemoral dynamics is critical in clinical practice. FHA is an essential functional property of a joint [15]. In the current study, we calculated the location and orientation of the continuous patellar FHA and found that it was close to, but not coincident with, the femoral TEA, and the PRR decreased slightly with knee flexion.

With the non-invasive, non-radiative and in vivo methodology based on static MR images used in this study, the patellar FHA was quantitatively demonstrated. Our previous study confirmed the reliability of static MR methodology in the establishment of a patellofemoral kinematics model [11]. Moreover, the current study was an in vivo study that included all soft tissues, which is closer to the actual physiological state than the two cadaver studies involving patellar FHA that are available to date [9, 10]. All intra- and inter-rater ICCs of the FHA parameters were greater than 0.93 (Table 2), indicating excellent repeatability of the TEA determination and the establishment of its coordinate system, which has a minor impact on the description of the location and orientation of the FHA.

The range and standard deviation of the patellar FHA varied with knee flexion angles. In the early and late stage, the variation ranges of all six parameters were larger than those in the middle stage, with a larger standard deviation among subjects. This was because the patella had not been captured by the trochlear groove and was only regulated by the soft tissue before 30° flexion [16], while in the late stage, the fluctuation of the FHA was attributed to the transformation of the patella moving away from the circular pathway in deep flexion as it moved onto the bilateral femoral condyles [9]. The variations in the FHA decreased in the middle stage, as a result of the femoral condyles enhancing patellar movement after it entered the trochlear groove [16]. Clinically, trochlear dysplasia is one of the common causes of patellar instability [17, 18]. Some realignment surgeries, including sulcus-deepening trochleoplasty and plication of the medial retinaculum, have been performed for decades [19]. However, the long-term outcome of these procedures is still debatable [20], which might be due to the insufficiency of restoring patellofemoral kinematics (including FHA) by surgery. Based on this hypothesis, patellofemoral motion models could be constructed for patients with patellar instability before an operation, and the computerised restoration of the appropriate patellar FHA would contribute to a precise surgical program. For example, accurate osteotomy depth and angle of the proximal trochlear groove could be calculated. Preoperative evaluation and virtual planning of patellofemoral kinematics would help to restore the normal motion of the patella and avoid post-operative patellar dislocation.

The patellar FHA presented L-shaped tracking in the midsagittal plane from the posteroinferior to the anterosuperior side of the TEA during knee flexion, differing in position and direction from the L-shaped tracking descripted in our previous study [12]. For example, the direction of the L-shaped tracking in this study looked oblique relative to the femoral shaft, but in our other study was upright. Coughlin et al. [10] stated that the origin of the patellar tracking arc was 9.6 mm anterior and 11.6 mm proximal to the TEA, close to the ultimate position of the middle stage in our study, but did not adequately evaluate the movement of the patellar FHA. The FHA translation was likely caused by diminishing radius of the trochlear groove curvature [21]. The decrease of this radius increased additional superior motion of the patella, which caused the FHA to shift anteriorly. This phenomenon is similar to the motion of tibiofemoral FHA previously reported by Sheehan et al. [7], who exemplified that an extra 1.1 mm of superior motion of the tibia during extension would result in a 25.0 mm posterior displacement of the tibiofemoral FHA.

In the early and middle stages, the PRR averaged 56.5 ± 10.9 mm, larger than that in previous studies (26.4–46.9 mm) [9, 10]. This is due to the different methods. For example, Coughlin et al. [9] chose the most prominent point on the dorsal ridge of the patella to fit the arc motion, differing from the patellar geometric centre selected in our study. From 20° to 30° flexion when the patella was captured by the trochlear groove, the average PRR showed a slight decrease, which was related to the slight diminishing radius of the trochlear groove [21]. Thus, it is critical to restore the trochlear groove’s geometry when designing prostheses. The trochlear groove’s geometry could be optimised by applying the curvature radius of the trochlear groove in a physiological state and the normal PRR in the middle stage of flexion, which is expected to reduce anterior knee pain after total knee arthroplasty.

In this study, the orientation of the patellar FHA was predominantly in a mediolateral direction, with an average A_F-T_ not exceeding 10° during most phases of knee flexion. However, at the initial stage of flexion, there were larger angles between the FHA and TEA, level plane, and coronal plane, which were likely due to the patella not following a circular path [9]. From full extension, the patella shifted medially with the guidance of the medial retinaculum [22], so that its FHA seemed to be oblique relative to the TEA, with the FHA orientation from superomedial to inferolateral. That is, medial shift of the patella increased A_F-T_ and A_F-L_. As the patellar shift slowed down, both A_F-T_ and A_F-L_ tended to decrease as well. Beyond 20° knee flexion, the patella shifted laterally in the trochlear groove [22, 23], causing a posterolateral-anteromedial orientation of the FHA. In the middle stage, A_F-L_ tended to decrease again with the patellar shift slowing down. In the late stage, the A_F-C_ transitioned to posterolateral-anteromedial orientation, possibly due to the slight medial shift of the patella [24].

To facilitate clinical application, we depicted the relationship between the FHA and the femoral TEA. Because the TEA is an anatomic marker which is easy to identify and is widely used clinically, and the present results showed that the patellar FHA was close to the TEA, it would be reliable to assess the FHA with the TEA as a reference. The insertion point of the medial and lateral retinaculum of the patellofemoral joint was near the sulcus of the medial epicondyle and the prominence of the lateral epicondyle, respectively [16], which explained why the FHA was close to the TEA. To date, the TEA has been regarded as an essential reference when installing the trochlear prosthesis in patellofemoral arthroplasty, and relatively successful clinical results have been achieved [25], which verifies the close relationship between the patellar FHA and TEA. Nevertheless, the FHA was nonoverlapping with the TEA, even if in the middle stage. This could be explained by the fact that the patella did not follow a complete circle that is influenced by the morphology of the trochlear groove, which resulted in the ever-changing FHA with knee flexion.

There were some limitations to this study. First, the patellar FHA was obtained under non-weight-bearing conditions without muscle loads, which might differ from that under weight-bearing conditions. Therefore, further studies about the effect of loading on the FHA are necessary. Second, the patellar motion calculated from finite static MR images was not completely congruent with the realistic one, but it was helpful for us to understand the spatial distribution of the patellar FHA; additionally, the accuracy of this method was confirmed in our previous study [11]. Third, due to the limited space of the MR machine, the maximum knee flexion angles of half of the 18 subjects were less than 110°, which could be improved by further equipment updates.

## Conclusions

In the current study, the location and direction of the continuous patellar FHA during knee flexion were quantitatively reported in detail. It was found that the average FHA drew L-shaped tracking on the midsagittal plane. The patellar FHA was close to, but not coincident with, the femoral TEA, both in location and orientation, and the patellar rotational radius decreased slightly with knee flexion. These findings could help us better understand the patellofemoral joint kinematics, and may provide directions for further studies on the differences in patellofemoral FHA among various types of patellofemoral disorders, thereby laying the foundation for the application of FHA in surgical evaluation, preoperative planning, and prosthesis design. All above is expected to promote the diagnosis and treatment of patellofemoral disorders.

## Supplementary Information

**Additional file 1.** Animation of the continuous motion process of the patella and the patellar FHA. (MP4 705 kb)

**Additional file 2.** The calculation formulas of patellar tracking and FHA.

**Additional file 3.** The curves of the six parameters of the FHA with knee flexion.

## Data Availability

The datasets used and/or analysed during the current study are available from the corresponding author on reasonable request.

## Abbreviations

3D: Three-dimensional
A_F-C_: Angles between patellar FHA and coronal plane
A_F-L_: Angles between patellar FHA and level plane
A_F-T_: Angles between patellar FHA and TEA
FHA: Finite helical axis
ICC: Intraclass correlation coefficient
IP: Intersection position between FHA and midsagittal plane
IP_y_: Y coordinate of IP
IP_z_: Z coordinate of IP
MR: Magnetic resonance
PRR: Patellar rotational radius
TEA: Trans-epicondylar axis
