# Supplementary material for: Relationship between patellofemoral finite helical axis and femoral trans-epicondylar axis using a static magnetic resonance-based methodology
Source: J Orthop Surg Res. 2021 Mar 24;16:212. doi: 10.1186/s13018-021-02328-2 (PMC7988974; doi:10.1186/s13018-021-02328-2)
Supplement: Supplementary file 2 — Additional file 2. The calculation formulas of patellar tracking and FHA. [file 13018_2021_2328_MOESM2_ESM.docx]

**Calculation of patellar tracking and FHA**

Continuous patellar tracking was calculated with the five knee models at five knee flexion angles using order-three spline algorithm. In brief, m non-collinear reference points of the patella were determined (m ≥ 3). The tracking of the reference points were derived from their coordinates at five angles of knee flexion with the order-three spline algorithm. Let radius vector P_i_(θ) denotes the tracking of the reference points (for i=1, 2, …, m), and θ is the knee flexion angle. The patellar motion is characterised by an orthogonal rotation matrix R(θ), and a translation vector v(θ). The Lagrangian function f was defined as:

$f(R\left( \theta\right),v(\theta))=\frac{1}{n}\sum_{i=1}^{m} {[R\left( \theta\right)*P_{i}\left( 0 \right)+v\left( \theta\right)-P_{i}\left( \theta\right)]}^{T}*\left[ R\left( \theta\right)*P_{i}\left( 0 \right)+v\left( \theta\right)-P_{i}\left( \theta\right) \right]$ (1)

The Lagrangian multiplier theorem was used to determine the R(θ) and v(θ) that minimise f under the constraint condition of rigid motion:

$R^{T}\left( \theta\right)* R\left( \theta\right)=I$ (2)

where I is the identity matrix. The accuracy of the calculated patellar tracking was validated with the precise tracking which was obtained from the motion capture experiment in our previous *in vitro* study.

Then, with the Chasles theorem, the FHA of the patellar motion was derived from patellar tracking with a 1° increment of knee flexion angle. Each FHA is represented as a function:

$Y\left( \theta\right)=s\left( \theta\right)+x*n\left( \theta\right)$ (3)

where n is the unit vector along the FHA, and s is the radius vector of a point on the FHA. s(θ) and n(θ) can be calculated from R(θ), R(θ+1°), v(θ), and v(θ+1°) by satisfying equations (4–6):

$R^{'}=R\left( \theta+1^{\circ} \right)R^{T}\left( \theta\right)$ (4)

$v'=v\left( \theta+1^{\circ} \right)-R^{'}v\left( \theta\right)$ (5)

$R^{'}w+v^{'}=w+tn\left( \theta\right)+\left( 1-cos\varphi\right)n\left( \theta\right)*\left( n\left( \theta\right)*\left( w-s\left( \theta\right) \right) \right)+sin\varphi n\left( \theta\right)*\left( w-s\left( \theta\right) \right)$(6)

where R’ and v’ are the rotational matrix and translation vector representing the patellar motion from θ to θ+1° knee flexion. t is the patellar translation along the FHA, and φ is the patellar rotation angle around the FHA. Equation (6) holds for any vector of w.
