# Supplementary figures and images for: Relationship between patellofemoral finite helical axis and femoral trans-epicondylar axis using a static magnetic resonance-based methodology
Source: J Orthop Surg Res. 2021 Mar 24;16:212. doi: 10.1186/s13018-021-02328-2 (PMC7988974; doi:10.1186/s13018-021-02328-2)

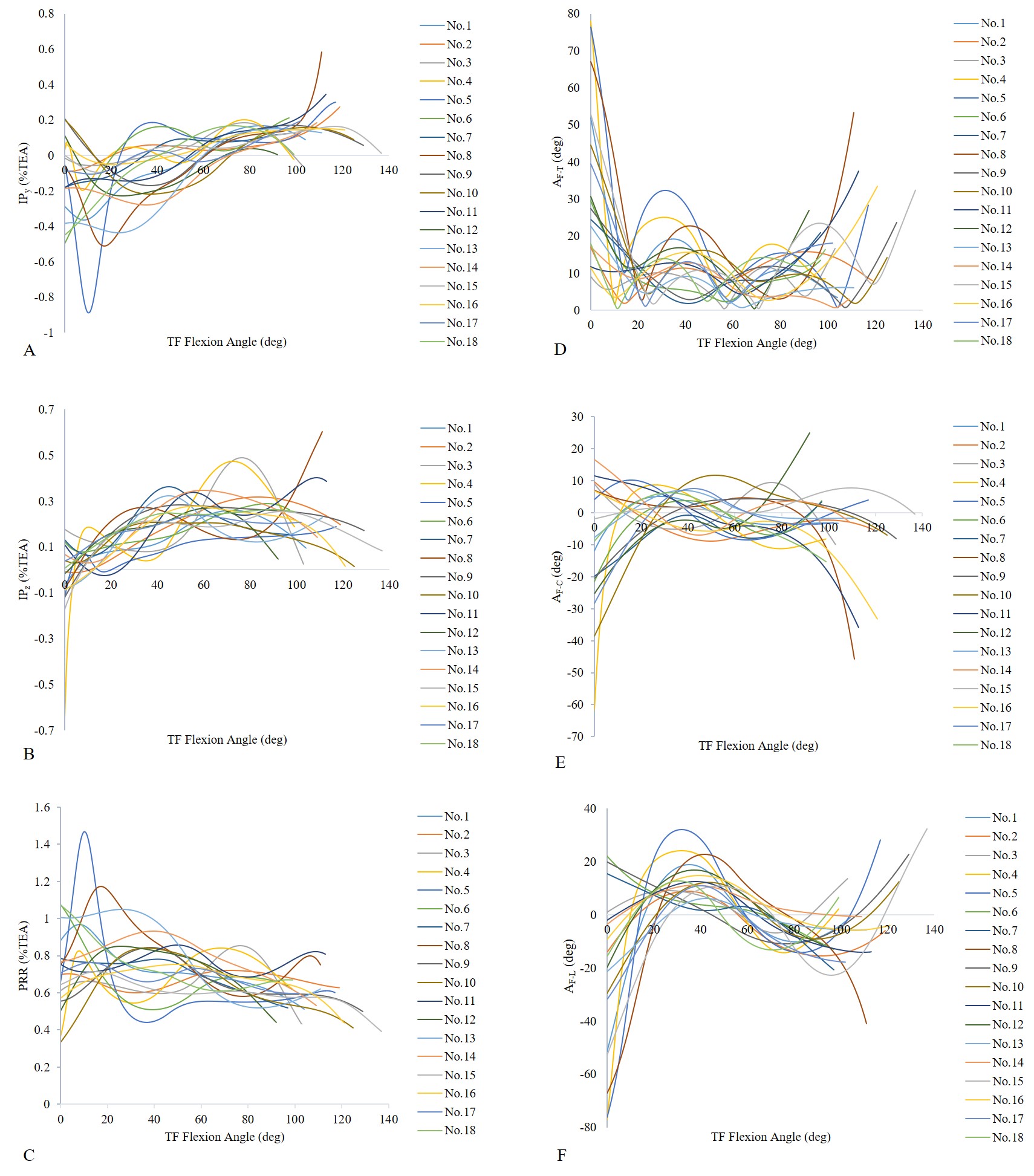

Supplement: Supplementary file 3 — Additional file 3. The curves of the six parameters of the FHA with knee flexion. [file 13018_2021_2328_MOESM3_ESM.jpg]
